# Effects of Modified Melatonin Release on Human Colostrum Neutrophils to Induce Death in the MCF-7 Cell Line

**DOI:** 10.1155/2022/8069188

**Published:** 2022-05-18

**Authors:** Waynner O. Sousa, Mahmi Fujimori, Tassiane C. Morais, Milena B. Santos, Gabriel F. S. Rodrigues, Katleyn P. G. Silva, André H. F. Torres, Adenilda C. Honorio-França, Eduardo L. França

**Affiliations:** Institute of Biological and Health Science, Federal University of Mato Grosso, Barra do Garças CEP 78600-000, Brazil

## Abstract

Cancer is one of the diseases with the highest mortality rate today, with breast cancer being the second most common type among the Brazilian population. Due to its etiological complexity and inefficiency of treatments, studies have focused on new forms of treatment. Among these forms of treatment, hormonal therapy seems to be an excellent auxiliary mechanism in tumoricidal activity, and melatonin has great potential as a modulator of the immune system. Thus, the present study is aimed at evaluating the effect of the hormone melatonin on the coculture of colostrum polymorphonuclear cells and MCF-7 cancer cells and evaluates the effect of this hormone using a modified transport system. A feasibility analysis was performed by fluorescence microscopy at three cell incubation times, 2 hours, 24 hours, and 72 hours. The measurement of cytokines in the cell supernatant occurred in 24 hours, and the apoptosis assay was performed in 72 hours using flow cytometry. The results showed higher levels of cell viability in groups treated with melatonin and less viability in groups containing a coculture of polymorphonuclear cells and MCF-7 after 72 hours of incubation. Furthermore, the apoptosis and necrosis rates were higher in coculture polymorphonuclear and MCF-7 cells, especially in groups containing microemulsion as a modified release agent. These data suggest that melatonin, especially if associated with a modified release system, has immunomodulatory effects on human colostrum polymorphonuclear cells. These cells can play a crucial role in the resolution of the tumor through their mediation and inflammatory action.

## 1. Introduction

Cancer development is a multifactorial process that involves genetic and environmental regulations. In addition, cancer cells have several unique characteristics that give resistance to the human immune system and cancer treatment. As a result, cancer treatment remains associated with several challenges [1, 2].

Breast cancer has the highest incidence in women and is diagnosed globally by physical examination, breast imaging, and tissue biopsies [3]. The most aggressive breast cancer is triple-negative because there are no receptors for estrogen (ER), progesterone (PR), and human epidermal growth factor receptor 2 (HER2), also called ER/PR- and HER2-. It is estimated that 70% of breast cancers are positive for hormone receptors, and both can be treated with complementary therapies but with different degrees of responsiveness [4–6].

Cancer treatment options include radiotherapy, chemotherapy, biological agent immunotherapy, and hormone therapy [5, 7]. In addition, the hormone melatonin has been identified as a possible agent for alternative immunotherapeutic treatment of cancer due to antimitotic activity [8, 9] and the modulatory capacity of the immune system [10–12].

Due to its systemic circulation, the melatonin hormone is identified in secretions [13]. In colostrum, we can find high melatonin levels at night and lower levels during the day, indicating a time-dependent bioavailability and fluctuation in the production of milk components [14, 15]. In addition, the modulatory activity in cells reduces the viability of cancer cells when in the presence of melatonin, especially when optimized with the use of biotechnology in modified systems, which suggests being a possible tool for alternative therapies for the treatment of cancer patients [12, 16–19].

Studies have pointed out fluctuations in serum melatonin concentrations depending on the stage of cancer. However, these differences are more related to biochemical changes than as a way of controlling neoplastic growth [20].

In addition to hormones, soluble bioactive components, and anti-infectious factors, human colostrum contains large amounts of viable leukocytes (10^9^ cells/ml in the first days of lactation), especially macrophages and neutrophils [21, 22]. Traditionally, researchers think of neutrophils only as agents in the acute stages of inflammation, and that they function only as exterminators of pathogens. However, recent findings have shown that neutrophil functions expand beyond roles in infection [23].

Some modified-release systems have been used in therapy for better delivery of melatonin due to their high metabolism and the ability of these therapies to guarantee their bioavailability, optimizing biological processes influenced by the neurohormone. Melatonin secretion occurs for 8 to 10 hours a day, with a maximum peak at approximately 3-4 hours in the morning. However, it exhibits a short half-life in the blood, with 80% excreted exclusively in the urine as 6-sulfatoxymelatonin in the first hour [24].

Thus, the modified release can be used as strategies for stimulating and/or inhibitory agents in various physiological processes, facilitating current treatments, reducing costs, side effects, increasing antitumor activities, and potentially could serve as a basis for future treatments.

Considering that milk, through cells, interacts with breast tissue and considering that melatonin, through the use of modified drug releasers, increases the functional activity of cells, it is possible that interactions between polymorphonuclear colostrum and the modified release melatonin have effects on breast tumor cells. Thus, the present study is aimed at evaluating the effect of the hormone melatonin on the coculture of colostrum polymorphonuclear cells and MCF-7 cancer cells and evaluates the effect of this hormone using a modified transport system.

## 2. Materials and Methods

One hundred twenty-four colostrum samples were collected from healthy mothers aged 18 and 40. After signing the free and informed consent form (attached), approximately 8 ml was collected by hand milking during the day during the first 48 to 72 hours postpartum, with an interval between two feedings. This work was approved by the Human Research Ethics Committee of the Araguaia under number 1,064,829.

### 2.1. Cell Separation

The samples were stored in a sterile plastic tube and centrifuged for 10 minutes at 160 G under refrigeration at 4°C, separating the colostrum into three distinct phases, cellular “button,” intermediate aqueous phase, and lipid supernatant, according to Honorio-França [21]. Next, the cell button was resuspended in 199 culture medium (Gibco) and separated in a density gradient with Ficoll-Paque (Pharmacia) for 40 minutes at 160 × g at a temperature of 4°C. Then, the cells were adjusted to a final concentration of 2 × 10^6^ cells/ml by light microscopy.

### 2.2. Tumor Cell Culture

MCF-7 human breast cancer cells were obtained from the American Type Culture Collection (ATCC, Manassas, VA, USA), frozen in liquid nitrogen for storage, thawed, and later grown in 75 cm^2^ plastic culture flasks in the middle Roswell Park Memorial Institute (RPMI) 1640 supplemented with 10% fetal bovine serum (Sigma-Aldrich Co., St Louis, MO, USA), penicillin (20 U/ml), and streptomycin (20 *μ*g/ml) (Sigma-Aldrich Co.) at 37°C in a humid atmosphere containing 5% CO_2_ until the formation of a cell monolayer. Then, trypsin was used to remove cell adhesion to the walls of the flasks. Finally, the cells were washed in phosphate buffer (PBS) and adjusted to a final concentration of 2 × 10^6^ cells/ml using optical microscopy.

### 2.3. Preparation of Microemulsion

The microemulsion was formulated with distilled water, caprylic/capric triglyceride-Polymol 812®, HLB = 10.8 (Emfal®, Betim, Brazil), oiled sorbitan-Span 80® (SP), HLB = 4.3, (Emfal®, Betim, Brazil), polysorbate 80-Tween 80® (TW)-HLB = 15.0, (Vetec®, Rio de Janeiro, Brazil), and 1-butanol (BT), (Vetec®, Rio de Janeiro, Brazil), according to Ribeiro et al. [25], and as a chemical reference substance, melatonin was used at a 100 ng/ml concentration.

### 2.4. Cell Viability

The cells were incubated at 37°C in culture medium 199 (Gibco) for 2, 24, and 72 hours, subsequently washed with PBS to remove the medium, and stained with the acridine orange vital dye. The percentages of viable cells and cells in the death process were determined using fluorescence microscopy.

To evaluate the cellular proliferation index in coculture (PMN and MCF-7 cells), it was incubated with propidium iodide (PI), and subsequent fluorescence detection allowed for assessment of the number of nonvital cells (first measurement). Thus, the cells were treated with melatonin incorporated or not to microemulsion and put in 24-well culture plates. The cells were maintained in culture for 72 hours, stained with PI, and had access to total DNA, leading to total cell population counts (second measurement). The fluorescence of the cells was analyzed by flow cytometry (FACSCalibur system; BD, San Jose, USA). The difference between these two measurements calculated the number of viable cells. The cellular proliferation index was calculated using the number of viable cells treated/number of viable cells not treated × 100 [26].

### 2.5. Cytokine Determination

Colostrum supernatant was collected and stored at -80°C before analysis. Then, the samples were thawed and incubated at 37°C in 199 medium for 72 hours, and the cytokines IL-6 and TNF-*α* were measured by Cytometric Bead Array (CBA, BD Biosciences, USA) according to the manufacturer's procedures. A flow cytometer was used for these analyses (FACSCalibur, BD/Biosciences, USA); cytometric plots were generated using CellQuest software (BD Biosciences, USA).

### 2.6. Apoptosis and Necrosis Assay

The sample groups were previously incubated at 37°C for 72 hours in the presence of 199 medium. According to the manufacturer's recommendations, the analysis of apoptosis and necrosis was performed using FITC Annexin V and propidium iodide (Sigma, St. Louis, USA). The cells were incubated for 10 min at room temperature. After that period, the cells were analyzed by flow cytometry (FACSCalibur, BD Biosciences, USA); cytometric plots were generated using CellQuest software (BD Biosciences, USA). The apoptotic cells were positive for Annexin V and negative for propidium iodide. The cells considered to be necrotic were positive for propidium iodide and negative for Annexin.

### 2.7. Statistical Analysis

The data are expressed as the mean ± standard deviation. Significant differences were evaluated using variance analysis (ANOVA one criterion) for viability, proliferation cells, and cytokines. They were compared at the level of significance at *p* < 0.05 by Bioestat v5.3 software. Another test (Tukey) was applied to determine the differences between treatments when the results showed statistically significant differences. Factorial ANOVA (*a* × *b*) test was used to determine the interaction between time treatments. For apoptosis and necrosis, the Kruskal-Wallis nonparametric test was used when statistical significance was found (*p* < 0.05). Finally, the Student-Newman-Keuls test was applied to determine the differences between treatments.

## 3. Results

### 3.1. Cell Viability

**Figure 1:**
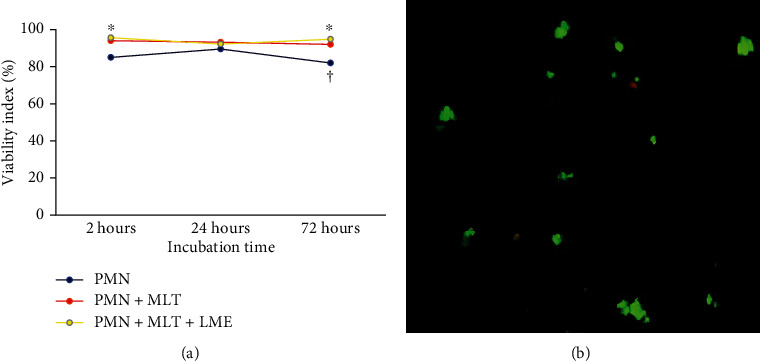
Time-dependent correlation of viability of PMN cells stimulated with melatonin (a). *F* = 9.26; *p* = 0.0018 (time of incubation), *F* = 14.4991; *p* = 0.0003 (treatment). The results represent the median of the standard deviation. PMN: polymorphonuclear cells; MLT: melatonin; LME: liquid microemulsion. The polymorphonuclear phagocytes were incubated with melatonin after 72 hours of incubation (a). Orange-stained cells (dead) and green-stained cells (alive). Experiments were repeated five times, and the results were comparable. *p* < 0.05. ^∗^Differences between PMN with treated PMN (MLT or MLT + LME), considering the same incubation times. †Differences between times of incubation, considering the same treatment.

The viability index was determined in three stages: 2 hours, 24 hours, and 72 hours of incubation, and the results are shown in Figure 1 and Table 1. The viability index decreased in colostrum PMN cells after 72 hours of incubation. Conversely, the treatment of cells with melatonin incorporated or not to microemulsion increased the viability index after 2 and 72 hours of incubation (Figure 1).

After 2 hours, the viability index showed a significant difference between the MCF-7 cells and the PMN cells. Regardless of treatment and incubation time, there were no significant differences in cell viability after 24 hours of incubation (*p* > 0.05). After 72 hours of incubation, the PMN cells treated with melatonin and coculture of PMN and MCF-7-cells treated with melatonin incorporated with microemulsion showed increased viability. In contrast, the coculture of PMN MCF-7 cells showed decreased viability (Table 1).

The lower cell proliferation index was observed in coculture of colostrum PMN cells incubated with melatonin incorporated or not to microemulsions (Table 2).

### 3.2. Cytokines

The cytokine levels are shown in Table 3. It was observed that independent of treatment, no differences in IL-6 concentrations in the supernatant of the PMNs cocultured or not cocultured with MCF-7 cells were observed. However, the TNF-*α* concentrations were lower in the supernatant of PMN cells cocultured or not with MCF-7 and incubated with melatonin (Table 3).

### 3.3. Apoptosis and Necrosis

**Figure 2:**
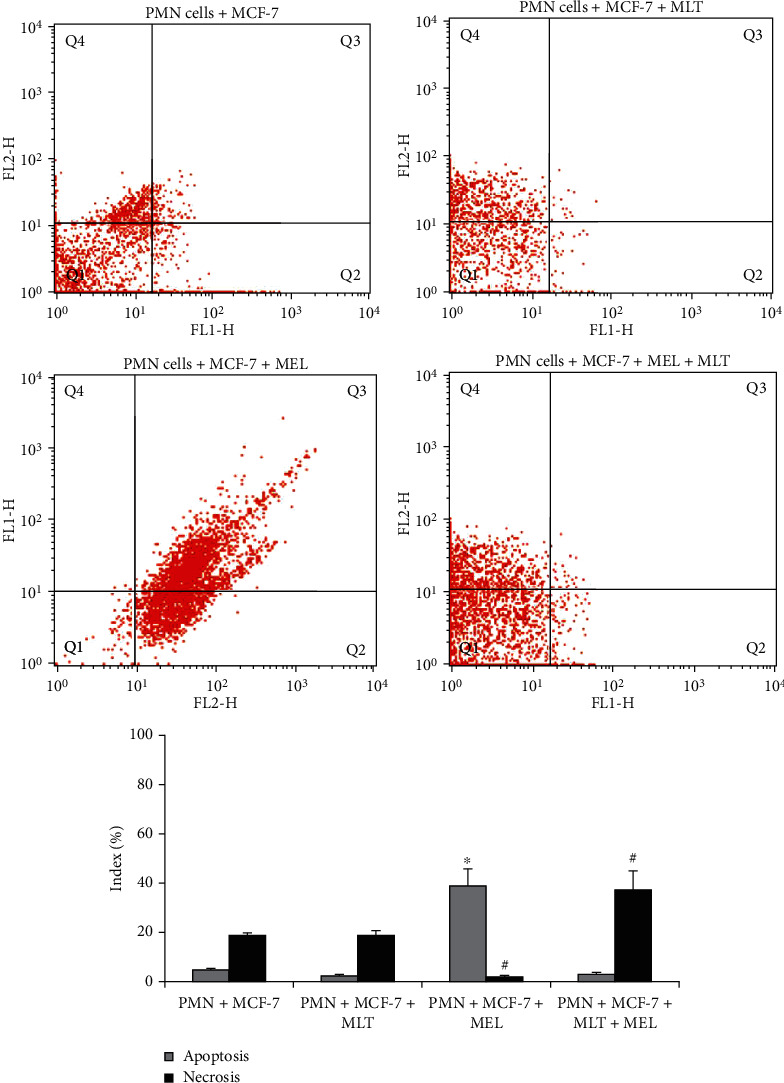
Apoptosis and necrosis in coculture of PMN cells and MCF-7 cells after 72 hours of incubation. PMN: polymorphonuclear cells; MCF-7: human breast cancer cells; MLT: melatonin; MEL: microemulsion. Cells were stained with Annexin V/PI and analyzed by flow cytometry. In the dot plot, the lower-left (Q1) quadrant corresponds to a viable cell; the lower-right (Q2) and upper-right (Q3) quadrants represent the percentage of apoptosis, and the upper-left (Q4) quadrant is the percentage of necrosis. *p* < 0.05. ^∗^Compared to apoptosis index of PMNs untreated with PMNs treated; ^#^compared to necrosis index of PMNs treated with PMNs.

**Figure 3:**
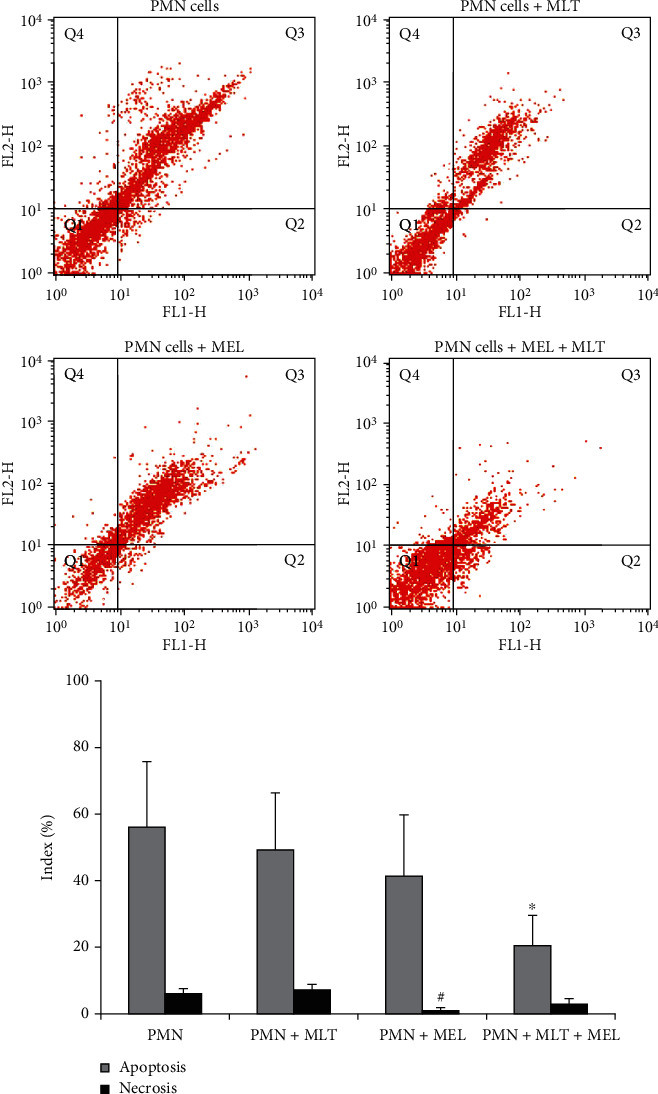
Apoptosis and necrosis in colostrum PMN cells. PMN: polymorphonuclear cells; MLT: melatonin; MEL: microemulsion after 72 hours of incubation. Cells were stained with Annexin V/PI and analyzed by flow cytometry. In the dot plot, the lower-left (Q1) quadrant corresponds to a viable cell; the lower-right (Q2) and upper-right (Q3) quadrants represent the percentage of apoptosis, and the upper-left (Q4) quadrant is the percentage of necrosis. *p* < 0.05. ^∗^Compared to apoptosis index of PMNs untreated with PMNs treated; ^#^compared to necrosis index of PMNs treated with PMNs.

In this work, it was also possible to compare the evaluation of cell death processes. The rates of apoptosis and necrosis in groups treated with melatonin and with the use of the modified system are available in Figure 2. The PMN cells treated with melatonin incorporated into the microemulsion had a lower apoptosis rate; similarly, coculture PMN and MCF-7 cells with and without MLT treatment had lower apoptosis rates, while the PMN treated with melatonin had lower rates of necrosis, as shown in Figure 3.

**Figure 4:**
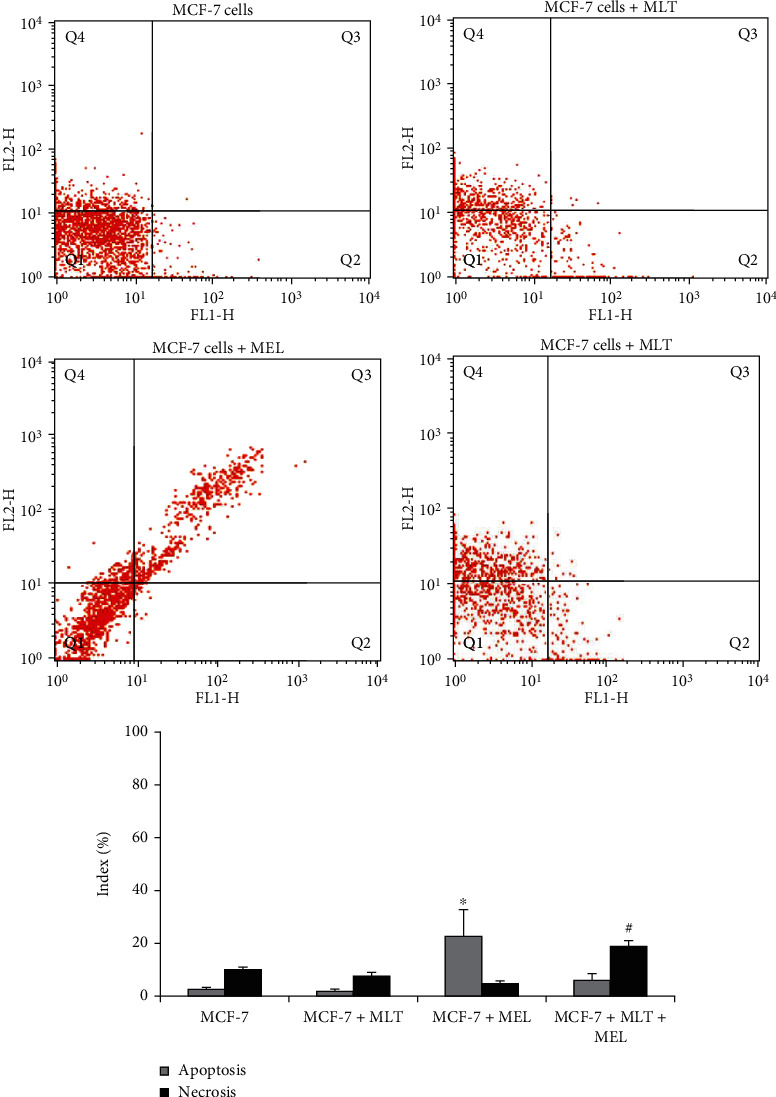
Apoptosis and necrosis indexes in MCF-7 cells. MCF-7: human breast cancer cells; MLT: melatonin; MEL: microemulsion after 72 hours of incubation. Cells were stained with Annexin V/PI and analyzed by flow cytometry. In the dot plot, the lower-left (Q1) quadrant corresponds to a viable cell; the lower-right (Q2) and upper-right (Q3) quadrants represent the percentage of apoptosis, and the upper-left (Q4) quadrant is the percentage of necrosis. *p* < 0.05. ^∗^Compared to apoptosis index of PMNs untreated with PMNs treated; ^#^compared to necrosis index of PMNs treated with PMNs.

The MCF-7 cells in the presence of the modified system release showed significant apoptosis rates compared to the untreated MCF-7 cells; however, when MCF-7 cells were treated with melatonin, there was a decrease in apoptosis rates and an increase in the necrosis index of these cells, as shown in Figure 4.

In addition, a higher rate of apoptosis was observed in the coculture group in the presence of the modified system release; however, when treated with melatonin and associated with the use of a modified system release, an increase in the rate of necrosis was observed, as shown in Figure 2.

## 4. Discussion

Phagocytes are the target of studies due to their ability to acquire different functionalities depending on the exposed stimuli. Some of these studies evaluated its phagocytic and apoptotic potential in the most various modulatory substances, where melatonin appears to be one of the main agents [17, 19, 25]. However, the hormone melatonin's immunostimulatory effects seem to be lower due to its antioxidative potential [27]. This study shows that colostrum PMN cells in the coculture of MCF-7 cells present increased apoptosis and necrosis rates, especially when treated with melatonin incorporated into the microemulsion.

The role of melatonin with and without nanoparticle-based delivery methods in promoting apoptosis in human breast cancer cells was related in other studies [28–31]. Still, none of them verified the interaction between colostrum neutrophils and breast cancer cells. Our experimental model employed colostral PMN cells and breast cancer cells to reproduce physiopathological conditions since the colostrum cells provide a good cancer research model [32], especially for breast cancer [33]. Colostrum cells are constituted by mononuclear (MN) and polymorphonuclear (PMN) cells; among them, neutrophils are cells whose effects are poorly studied [21, 34]. Previous studies confirm that the high concentration of phagocytes in the colostrum is probably responsible for initiating different killing mechanisms [32] and inducing apoptosis in tumor cells [35].

Melatonin acts as an immunomodulatory agent in PMN phagocytes, and variations in serum melatonin levels can influence how phagocytes respond to the stimulus. It was also observed that both cell types, mononuclear (MN) and polymorphonuclear, had increased phagocytosis in the presence of the hormone, both in daytime and nighttime samples; however, PMN had a lower bactericidal rate in night-time samples in the presence of melatonin, that showed the stimulating effect of melatonin on phagocytes and that melatonin stimulates the oxidative metabolism of phagocytic cells, against EPEC [19]. In this study, we observed that cell viability in culture evaluated by acridine orange is time-dependent, and that melatonin improved PMN cell viability at all incubation times. The acridine orange is a vital metachromatic fluorochrome dye that binds to cellular DNA or RNA [36]. When examined under an ultraviolet microscope, it emits a green, orthochromatic color in contact with double-stranded DNA. When in contact with denatured or depolarized DNA, or single-stranded RNA, it emits an orange or red color in the metachromatic form [37].

Studies have demonstrated the role of melatonin in preventing the death of human leukocytes [38]. Here, despite the incubation time, melatonin maintained viable colostrum cells. Furthermore, the melatonin effects are dose-dependent, wherein at lower concentrations, this hormone presents effects of cellular activation, whereas in higher doses, the melatonin show cellular inhibitory effects [19, 39–41].

The capacity of phagocytes to act on cancer cells remains unresolved. Nevertheless, there is evidence that PMNs effectively prevent cancer and are associated with cancer control [28]. Additionally, various immunostimulatory molecules have been associated with antitumor effectors, such as cytokines. Evidence indicates that serum levels of IL-6 and TNF-*α* may be elevated in several cancers [27, 42, 43], and both proinflammatory cytokines present dichotomous since they have function that maintains the body's homeostasis. Still, at the same time, they may take on a protumoral role [43]. In this work, we have demonstrated that the culture supernatant of colostrum PMN cells and MCF-7 cells, in the presence of melatonin, decreased TNF-*α* levels.

The role of TNF-*α* in the promotion and inhibition of tumors still seems uncertain; however, it seems certain that melatonin can inhibit the expression of this cytokine present in the inflammatory process. The present research also made it possible to compare the evaluation of cell death processes through apoptosis and necrosis, which are essential for the development and homeostasis of tissues, participating in the immune response, and, in general, in all pathophysiological processes [44].

Studies have related the role of melatonin in apoptosis in numerous types of cells; among these studies, it is possible to highlight the death of cancer cells, including human hepatocarcinoma of the HEPG2 lineage and tumor line MCF-7 of mammary adenocarcinoma [12, 45–47], however, in some studies, melatonin also has antioxidative potential for oncotic cells [27].

Apoptosis, unlike necrosis, in most cases, does not trigger an inflammatory response, which makes the process more attractive to studies looking for alternative treatments for tumor cell death [44].

Studies have shown that various environmental stimuli can initiate or inhibit physiological and pathological apoptosis [48]. Thus, cell death occurs for every organism due to the most varied physiological processes, which could justify high apoptosis rates in groups of PMN cells.

However, it is natural that they survive in circulation for approximately 7-10 h. Still, when inflammatory substances or chemicals stimulate cells, they can remain active for 48 hours [23]. This fact would explain the high apoptosis rates in the control groups of polymorphonuclear cells found in this work.

The neutrophil is a key effector cell at the forefront of immune defense and is effective in most cases due to its versatility. Neutrophils prove to be effective phagocytes because, when performing this task, they are programmed to die with an immediate, silent, and importantly contained apoptotic death [23].

Apoptosis occurs spontaneously in untreated malignancies and participates in at least some types of therapy-induced tumor regression. In addition, it is involved in physiological involution and atrophy of various tissues and organs [49].

Some of these studies suggest that apoptosis and necrosis continue the same cell death process. Their implications and forms for neighboring tissues would be decided by the availability of ATP and probably by additional factors in the dying and eliminator cells [49]. Two distinct apoptotic processes are triggered by melatonin in MCF-7 cells: one form is apoptosis independent of caspase associated with an increase in the p53/MDM2 protein ratio (responsible for cell growth/apoptosis induction), increased release of apoptotic induced factor (AIF), and no change in caspase or cleaved activity; the second is a late apoptotic process, dependent on TGF1 [50, 51].

Considering that the induction of macrophage apoptosis by pathogenic agents can negatively affect the host's immune response to infection, the acceleration of the apoptotic process of neutrophils after phagocytic interaction with bacteria seems essential for the resolution of the infection. This idea is supported by discovering that some bacterial pathogens alter neutrophil apoptosis induced by normal phagocytosis to survive and cause disease. However, although progress has been made toward understanding apoptosis in neutrophils, very little is known about the transcriptional regulation of this process during bacterial infection [52, 53].

Apoptotic PMNs cannot remain that way forever, and if phagocytes do not eliminate them, they will undergo necrosis if all good work is undone. Large-scale recruitment of PMNs must necessarily be followed by apoptosis and subsequent removal by macrophages to resolve inflammation [54, 55].

Although the term “programmed cell death” has been commonly associated with apoptosis, recent studies have proposed that, under specific conditions, necrosis represents a regulated and well-orchestrated process [56]. However, necrosis has always been considered almost “accidental” cell death, a random and uncontrolled process [57–59]. In addition, it was also possible to observe an increase in necrosis rates in the coculture of PMN cells and MCF-7 cells, with its highest rates in the presence of melatonin incorporated in the microemulsion.

Programmed necrosis is cell death independent of caspase and is always triggered as a backup mechanism for apoptosis when caspases are inactivated. In addition, programmed necrosis is accompanied by autophagy, but the specific relationship remains an enigma [60].

Animal studies in maintaining mammary gland homeostasis and cell death, specifically of the PMN, are of great importance. Despite being essential as the first line of cellular defense against mastitis pathogens [61], PMNs have great potential to destroy glandular tissue, either by the secretion of inflammatory mediators or by cytoplasmic leakage resulting from necrosis. In addition, there is an increase in the percentage of this cell type by up to 90% [62].

Another phenomenon also described is the necrosis of apoptotic bodies that took time to be phagocytosed by macrophages and removed from the inflamed site [63]. This event may contribute to the increase in necrosis observed in the present study.

In this study, a higher rate of necrosis and apoptosis was observed in cells cocultured in the presence of melatonin, and an additional anticancer effect was demonstrated when melatonin was incorporated into the microemulsion. In addition, studies indicate that melatonin adsorbed on PEG microspheres has antitumor effects on MCF-7 human breast cancer cells [12]. However, these effects are not restricted to MCF-7 cells. For example, a study with lung tumors showed that MEs exhibited an effect, decreasing cytotoxicity, inducing apoptosis, and inhibiting tumor growth and survival time [64].

Melatonin acts through a receptor, activating signaling pathways involving second messengers cAMP, diacylglycerol, inositol triphosphate (IP-3), and intracellular Ca2+ [42, 43]. The release of large amounts of intracellular Ca2+ has been associated with the induction of apoptosis in human cells when in the presence of tumor cells [47, 65].

Also, studies have associated microemulsion as a carrier system capable of altering the intracellular influx of Ca2+ in human cells, which can lead to cellular damage that culminates in the activation of cell death pathways, such as apoptosis, which may explain in this study the high levels of apoptosis when in the presence of microemulsion and depending on the dose can increase or reduce necrosis [66].

It should be considered that breast tissue is in constant and direct contact with milk's soluble and cellular immunological components, among these high concentrations of melatonin and PMN phagocytes. Therefore, the interactions of these components through modified delivery systems, increasing the stability of melatonin, can probably be an alternative for tumor immunotherapy [66].

## 5. Conclusions

The data suggest that melatonin has time-dependent immunomodulatory effects on colostrum polymorphonuclear cells, increasing the viability of cells. In addition, melatonin demonstrated the ability to decrease the TNF-*α* level in the supernatant of PMN and MCF-7 cell cultures, proving to be a potent anti-inflammatory agent. Melatonin also, in the culture of PMNs and cells, was able to increase cell death by necrosis, and the use of microemulsion enhanced this effect. In this way, the findings of this research allowed us to verify that the therapies that use melatonin can be considered a viable alternative to explore new studies that characterize the mechanisms of action of polymorphonuclear cells in synergy with the melatonin hormone.

## Figures and Tables

**Table 1:** Viability index of colostrum polymorphonuclear cells (PMNs) and MCF-7 cells treated or not with melatonin at different incubation times.

	Time in hours		
	2	24	72
PMN	95.6 ± 1.7	90.4 ± 2.6	83.2 ± 4.2^#^
MCF-7	91.6 ± 2.6^∗^	91.2 ± 4.1	80.8 ± 3.3^#^
PMN + MCF-7	96.0 ± 3.2	92.0 ± 2.0	74.8 ± 11.5^∗^^#^
PMN + MLT	94.0 ± 3.2	93.2 ± 1.8	92.0 ± 2.5^∗^
MCF-7 + MLT	94.0 ± 4.0	90.0 ± 2.8	79.6 ± 10.0
PMN + MCF-7 + MLT	94.8 ± 2.3	88.4 ± 4.6	80.0 ± 8.8^#^
PMN + MCF-7 + MLT + MEL	95.6 ± 3.7	92.2 ± 4.1	94.8 ± 2.1^∗^

Notes: the results represent the median of the standard deviation of 10 experiments with different cells. PMN: polymorphonuclear cells; MCF-7: human breast cancer cells; MLT: melatonin; MEL: microemulsion. ^∗^*p* < 0.05: comparing treated and nontreated cells (RPMI 1640 medium), considering the same incubation time; #*p* < 0.05 comparing the incubation time, considering the same treatment.

**Table 2:** Cellular proliferation index of MCF-7 cells and cocutlure (PMN cells + MCF-7 cells).

	MCF-7 cells (%)	Coculture (%)
Medium	95.20 ± 2.8	96.1 ± 2.7
Melatonin	52.7 ± 2.81^∗^	33.4 ± 4.2^∗^^#^
MLT + MEL	43.6 ± 7.6^∗^	29.7 ± 1.3^∗^^#^

Note: PMN colostral polymorphonuclear cells; MCF-7: human breast cancer cells; MLT: melatonin; MEL: microemulsion. The results represent median of the standard and of five experiments with different cells. ^∗^*p* < 0.05, comparing treated and untreated cells. ^#^*p* < 0.05, comparing MCF-7 cells and coculture (MN and MCF-7 cells).

**Table 3:** IL-6 and TNF-*α* concentrations (pg/ml) in the culture supernatant of colostrum PMN cells cocultured or not with MCF-7 cells after 72 hours of incubation.

Cells	IL-6	TNF-*α*
PMN	4.13 ± 0.71	6.69 ± 1.42
MCF-7	4.31 ± 0.94	6.82 ± 1.18
PMN + MCF-7	3.80 ± 0.45	5.88 ± 1.01
PMN + MLT	4.27 ± 0.96	5.59 ± 0.42^∗^
MCF-7 + MLT	3.90 ± 0.45	5.58 ± 0.47^∗^
PMN + MCF-7 + MLT	3.89 ± 0.55	5.59 ± 0.56^∗^
PMN + MCF-7 + MLT + MLT	4.21 ± 0.26	5.01 ± 1.01^∗^

Notes: the results represent the median of the standard deviation of 10 experiments with different cells. PMN: polymorphonuclear cells; MCF-7: human breast cancer cells; MLT: melatonin. ^∗^*p* < 0.05: comparing treated and nontreated cells (RPMI 1640 medium).

## Data Availability

The original data shown in Tables 1–3 and Figures 1–4 used to support the findings of this study are available from the corresponding author upon request.

## References

[1] Hanahan D., Weinberg R. A. (2011). Hallmarks of cancer: the next generation. *Cell*.

[2] Su S.-C., Hsieh M.-J., Yang W.-E., Chung W.-H., Reiter R. J., Yang S.-F. (2017). Cancer metastasis: mechanisms of inhibition by melatonin. *Journal of Pineal Research*.

[3] Bray F., Ferlay J., Soerjomataram I., Siegel R. L., Torre L. A., Jemal A. (2018). Global cancer statistics 2018: GLOBOCAN estimates of incidence and mortality worldwide for 36 cancers in 185 countries. *CA: a Cancer Journal for Clinicians*.

[4] Weigelt B., Reis-Filho J. S. (2010). Molecular profiling currently offers no more than tumour morphology and basic immunohistochemistry. *Breast Cancer Research*.

[5] Gadi V. K., Davidson N. E. (2017). Practical approach to triple-negative breast cancer. *Journal Oncology Practice*.

[6] Waks A. G., Winer E. P. (2019). Breast cancer treatment. *JAMA*.

[7] Barrett S. V. (2010). Breast cancer. *Royal College of Physicians of Edinburgh*.

[8] Bindoni M., Jutisz M., Ribot G. (1976). Characterization and partial purification of a substance in the pineal gland which inhibits cell multiplication in vitro. *Biochimica et Biophysica Acta*.

[9] Fitzgerald T. J., Veal A. (1976). Melatonin antagonizes colchicine-induced mitotic arrest. *Experientia*.

[10] Reinaque A. P. B., França E. L., Scherer E. F., Côrtes M. A., Souto F. D. J., Honorio-França A. C. (2012). Natural material adsorbed onto a polymer to enhance immune function. *Drug Design, Development and Therapy*.

[11] Lin G.-J., Huang S.-H., Chen S.-J., Wang C.-H., Chang D.-M., Sytwu H.-K. (2013). Modulation by melatonin of the pathogenesis of inflammatory autoimmune diseases. *International Journal of Molecular Sciences*.

[12] França E. L., Honorio-França A. C., Fernandes R. T. S., Marins C. M. F., Pereira C. C. S., Varotti F. P. (2016). The effect of melatonin adsorbed to polyethylene glycol microspheres on the survival of MCF-7 cells. *Neuroimmunomodulation*.

[13] Stuebe A. (2009). The risks of not breastfeeding for mothers and infants. *Reviews in Obstetrics and Gynecology*.

[14] Illnerová H., Buresová M. (1993). Melatonin rhythm in human milk. *The Journal Clinical Endocrinology and Metabolism*.

[15] França E. L., Nicomedes T. D. R., Calderon I. D. M. P., Honorio-França A. C. (2010). Time-dependent alterations of soluble and cellular components in human milk. *Biological Rhythm Research*.

[16] Guimarães P. C. L., Honorio-França A. C., Hara C. D. C. P., Fagundes D. L. G., Ratto S. H. B., França E. L. (2013). Modulation of human colostrum phagocyte activity by the glycine-adsorbed polyethylene glycol microspheres. *Journal of Chemistry*.

[17] Hara C. D. C. P., Honorio-França A. C., Fagundes D. L. G., Guimarães P. C. L., França E. L. (2013). Melatonin nanoparticles adsorbed to polyethylene glycol microspheres as activators of human colostrum macrophages. *Journal of Nanomaterials*.

[18] Honorio-França A., Pessoa R., França E. (2015). Microemulsion of babaçu oil as a natural product to improve human immune system function. *Drug Design, Development and Therapy*.

[19] Honorio-França A. C., Hara C. C. P., Ormonde J. V. S., Nunes G. T., França E. L. (2013). Human colostrum melatonin exhibits a day-night variation and modulates the activity of colostral phagocytes. *Journal of Applied Biomedicine*.

[20] Dogliotti L., Berruti A., Buniva T. (1990). Melatonin and human cancer. *Journal of Steroid Biochemistry and Molecular Biology*.

[21] Honório-França A. C., Carvalho M. P. S. M., Isaac L., Trabulsi L. R., Carneiro-Sampaio M. M. S. (1997). Colostral mononuclear phagocytes are able to kill enteropathogenic Escherichia coli opsonized with colostral IgA. *Scandinavian Journal of Immunology*.

[22] Fagundes D. L. G., França E. L., Morceli G., Rudge M. V. C., Calderon I. D. M. P., Honorio-França A. C. (2013). The role of cytokines in the functional activity of phagocytes in blood and colostrum of diabetic mothers. *Clinical and Developmental Immunology*.

[23] Leitch A. E., Duffin R., Haslett C., Rossi A. G. (2008). Relevance of granulocyte apoptosis to resolution of inflammation at the respiratory mucosa. *Mucosal Immunology*.

[24] Quera-Salva M.-A., Claustrat B. (2018). Melatonin: physiological and pharmacological aspects related to sleep: the interest of a prolonged-release formulation (Circadin^®^) in insomnia. *L’Encephale*.

[25] Ribeiro E. B., Lanes P. K. D., Chaud N. G. A., Pessoa R. S., Honorio-França A. C., França E. L. (2015). Microemulsions with levamisole delivery systems as novel Immunomodulating agents with potential for amebiasis therapies. *Science of Advanced Materials*.

[26] Ribeiro A. A. L., Silva F. H., Cotrim A. C. M. (2018). Herbal Mixture Adsorbed to Polyethylene Glycol Microspheres Induces Apoptotic Effects on Breast Cancer Cells. *Current Drug Delivery*.

[27] Ognjanovic S., Yamamoto J., Saltzman B. (2010). Serum CRP and IL-6, genetic variants and risk of colorectal adenoma in a multiethnic population. *Cancer Causes and Control*.

[28] Priano L., Esposti D., Esposti R. (2007). Solid lipid nanoparticles incorporating melatonin as new model for sustained oral and transdermal delivery systems. *Journal Nanoscience Nanotechnology*.

[29] Gatti G., Lucini V., Dugnani S. (2017). Antiproliferative and pro-apoptotic activity of melatonin analogues on melanoma and breast cancer cells. *Oncotarget*.

[30] Chuffa L. G. A., Seiva F. R. F., Novais A. A. (2021). Melatonin-loaded nanocarriers: new horizons for therapeutic applications. *Molecules*.

[31] Tran Q. H., Hoang D. H., Song M. (2021). Melatonin and doxorubicin synergistically enhance apoptosis via autophagy- dependent reduction of AMPK*α*1 transcription in human breast cancer cells. *Experimental & Molecular Medicine*.

[32] Otten M. A., Rudolph E., Dechant M. (2005). Immature neutrophils mediate tumor cell killing via IgA but not IgG Fc receptors. *The Journal of Immunology*.

[33] Honorio-França A. C., Launay P., Carneiro-Sampaio M. M. S., Monteiro R. C. (2001). Colostral neutrophils express Fc alpha receptors [CD89] lacking gamma chain association and mediate noninflammatory properties of secretory IgA. *Journal of Leukocytes Biology*.

[34] Honorio-França A. C., Fernandes R. T. S., Tozetti I. A. (2021). Mechanism anti-tumor of IgA-based delivery system on the human colostral mononuclear cells via Fc*α* receptor. *Biointerface Research in Applied Chemistry*.

[35] Stockmeyer B., Beyer T., Neuhuber W. (2003). Polymorphonuclear granulocytes induce antibody-dependent apoptosis in human breast cancer cells. *Journal of Immunology*.

[36] Smith D. L., Rommel F. A. (1977). A rapid micro method for the simultaneous determination of phagocytic- microbiocidal activity of human peripheral blood leukocytes in vitro. *Journal of Immunological Methods*.

[37] Zelenin A. V., Maison W. T. (1999). Acridine Orange as a Probe for Cell and Molecular Biology. *Fluorescent and Luminescent Probes for Biological Activity*.

[38] Espino J., Bejarano I., Redondo P. C. (2010). Melatonin reduces apoptosis induced by calcium signaling in human leukocytes: evidence for the involvement of mitochondria and Bax activation. *Journal of Membrane Biology*.

[39] Ianãs O., Olinescu R., Bãdescu I. (1991). Melatonin involvement in oxidative processes. *Journal of Endocrinology*.

[40] Pandi-Perumal S. R., Trakht I., Srinivasan V. (2008). Physiological effects of melatonin: role of melatonin receptors and signal transduction pathways. *Progress in Neurobiology*.

[41] Morceli G., Honório-França A. C., Fagundes D. L. G., Calderon I. M. P., França E. L. (2013). Antioxidant effect of melatonin on the functional activity of colostral phagocytes in diabetic women. *PLoS One*.

[42] Kim S., Keku T. O., Martin C. (2008). Circulating levels of inflammatory cytokines and risk of colorectal adenomas. *Cancer Research*.

[43] Sasaki Y., Takeda H., Sato T. (2012). Serum interleukin-6, insulin, and HOMA-IR in male individuals with colorectal adenoma. *Clinical Cancer Research*.

[44] Fink S. L., Cookson B. T. (2005). Apoptosis, pyroptosis, and necrosis: mechanistic description of dead and dying eukaryotic cells. *Infection and Immunity*.

[45] Carbajo-Pescador S., García-Palomo A., Martín-Renedo J., Piva M., González-Gallego J., Mauriz J. L. (2011). Melatonin modulation of intracellular signaling pathways in hepatocarcinoma HepG2 cell line: role of the MT1 receptor. *Journal of Pineal Research*.

[46] Sainz R. M., Mayo J. C., Rodriguez C., Tan D. X., Lopez-Burillo S., Reiter R. J. (2003). Melatonin and cell death: differential actions on apoptosis in normal and cancer cells. *Cellular and Molecular Life Sciences*.

[47] Honorio-França A. C., Nunes G. T., Fagundes D. L. G. (2016). Intracellular calcium is a target of modulation of apoptosis in MCF-7 cells in the presence of IgA adsorbed to polyethylene glycol. *Oncotargets and Therapy*.

[48] Hengartner M. O. (2000). The biochemistry of apoptosis. *Nature*.

[49] Leist M., Single B., Castoldi A. F., Kühnle S., Nicotera P. (1997). Intracellular adenosine triphosphate (ATP) concentration: a switch in the decision between apoptosis and necrosis. *The Journal of Experimental Medicine*.

[50] Cucina A., Proietti S., D’Anselmi F. (2009). Evidence for a biphasic apoptotic pathway induced by melatonin in MCF-7 breast cancer cells. *Journal of Pineal Research*.

[51] Hitomi J., Katayama T., Eguchi Y. (2004). Involvement of caspase-4 in endoplasmic reticulum stress-induced apoptosis and A*β*-induced cell death. *The Journal of Cell Biology*.

[52] DeLeo F. R. (2014). Modulation of phagocyte apoptosis by bacterial pathogens. *Apoptosis*.

[53] Kobayashi S. D., Braughton K. R., Whitney A. R. (2003). Bacterial pathogens modulate an apoptosis differentiation program in human neutrophils. *Proceedings of the National Academy of Sciences*.

[54] Bianchi S. M., Dockrell D. H., Renshaw S. A., Sabroe I., Whyte M. K. B. (2006). Granulocyte apoptosis in the pathogenesis and resolution of lung disease. *Clinical Science*.

[55] Rossi A. G., Hallett J. M., Sawatzky D. A., Teixeira M. M., Haslett C. (2007). Modulation of granulocyte apoptosis can influence the resolution of inflammation. *Biochemical Society Transactions*.

[56] Kroemer G., Galluzzi L., Vandenabeele P. (2009). Classification of cell death: recommendations of the Nomenclature Committee on Cell Death 2009. *Cell Death and Differentiation*.

[57] Galluzzi L., Kroemer G. (2008). Necroptosis: a specialized pathway of programmed necrosis. *Cell*.

[58] Golstein P., Kroemer G. (2007). Cell death by necrosis: towards a molecular definition. *Trends in Biochemical Sciences*.

[59] Proskuryakov S. Y., Konoplyannikov A. G., Gabai V. L. (2003). Necrosis: a specific form of programmed cell death?. *Experimental Cell Research*.

[60] Degterev A., Huang Z., Boyce M. (2013). Addendum: chemical inhibitor of nonapoptotic cell death with therapeutic potential for ischemic brain injury. *Nature Chemical Biology*.

[61] Piepers S., Opsomer G., Meyer E. (2009). Heifer and quarter characteristics associated with periparturient blood and milk neutrophil apoptosis in healthy heifers and in heifers with subclinical mastitis. *Journal of Dairy Science*.

[62] Koess C., Hamann J. (2008). Detection of mastitis in the bovine mammary gland by flow cytometry at early stages. *Journal of Dairy Research*.

[63] Zhao X., Lacasse P. (2008). Mammary tissue damage during bovine mastitis: causes and control1. *Journal of Animal Science*.

[64] Qu D., Guo M., Qin Y. (2017). A multicomponent microemulsion using rational combination strategy improves lung cancer treatment through synergistic effects and deep tumor penetration. *Drug Delivery*.

[65] Fernandes R. T., França E. L., Triches D. L. (2019). Nanodoses of melatonin induces apoptosis on human breast cancer cells cocultured with colostrum cells. *Biointerface Research in Applied Chemistry*.

[66] Ham B., Fernandez M. C., D’Costa Z., Brodt P. (2016). The diverse roles of the TNF axis in cancer progression and metastasis. *Trends in Cancer Research*.

